# Alternative Splicing of MAPKs in the Regulation of Signaling Specificity

**DOI:** 10.3390/cells10123466

**Published:** 2021-12-08

**Authors:** Galia Maik-Rachline, Inbal Wortzel, Rony Seger

**Affiliations:** Department of Biological Regulation, Weizmann Institute of Science, Rehovot IL-7610001, Israel; galia.maik-rachline@weizmann.ac.il (G.M.-R.); inw2001@med.cornell.edu (I.W.)

**Keywords:** MAPK, alternative splicing, ERK, JNK, p38, ERK1c

## Abstract

The mitogen-activated protein kinase (MAPK) cascades transmit signals from extracellular stimuli to a variety of distinct cellular processes. The MAPKKs in each cascade specifically phosphorylate and activate their cognate MAPKs, indicating that this step funnels various signals into a seemingly linear pathway. Still, the effects of these cascades vary significantly, depending on the identity of the extracellular signals, which gives rise to proper outcomes. Therefore, it is clear that the specificity of the signals transmitted through the cascades is tightly regulated in order to secure the desired cell fate. Indeed, many regulatory components or processes that extend the specificity of the cascades have been identified. Here, we focus on a less discussed mechanism, that is, the role of distinct components in each tier of the cascade in extending the signaling specificity. We cover the role of distinct genes, and the alternatively spliced isoforms of MAPKKs and MAPKs, in the signaling specificity. The alternatively spliced MEK1b and ERK1c, which form an independent signaling route, are used as the main example. Unlike MEK1/2 and ERK1/2, this route’s functions are limited, including mainly the regulation of mitotic Golgi fragmentation. The unique roles of the alternatively spliced isoforms indicate that these components play an essential role in determining the proper cell fate in response to distinct stimulations.

## 1. Introduction

**Figure 1:**
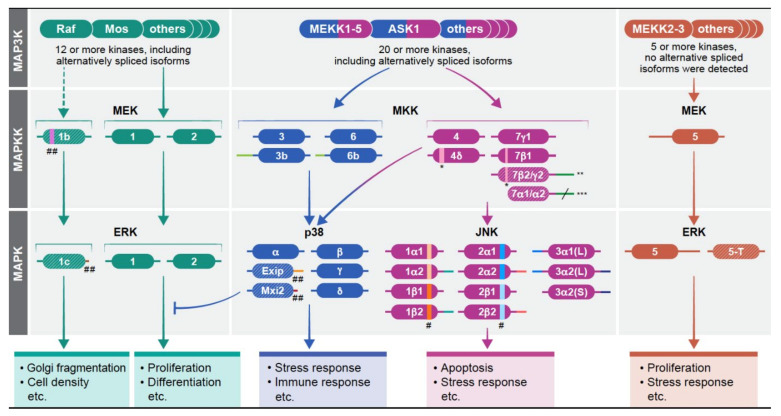
Signaling specificity and multiple isoforms of the MAPK signaling pathways. The MAPKs operate within signaling cascades composed of three to five layers (tiers) of protein kinases. The signals from the cascades are transmitted via sequential phosphorylation and activation of the components in each layer. The four cascades, which have been identified are shown: the human ERK1/2 cascade with MEK1/2/1b and ERK1/1c/2 at the MAPKK and MAPK layers. The p38MAPK cascade with MKK3/3b/6/6b, and p38α/Exip/Mxi2/p38β/p38γ/p38δ; the JNK cascade with MKK4/4δ/7γ1/γ2/β1/β2/α1/α2 and JNK1α1/1α2/1β1/1β2/2α1/2α2/2β1/2β2/3α1(L)/3α2(L)/3α2(S); and the ERK5 cascade with MEK5, and ERK5/5-T. It should be noted that the isoforms presented are those whose expression has been confirmed. Other alternatively spliced transcripts whose protein expression is not confirmed or may exist in other organisms are not presented here as well. The main proteins in each layer of each cascade (except for JNK) appear on top. As for JNK, it seems that all components may be substantially expressed, at least in some cells. Each of the kinases is composed of a kinase domain (central region) as well as N and *C* terminus represented by a line on the left (*N* terminus) and right (*C* terminus) of all kinase domains. The patterns in some of the proteins (e.g., MEK1b) represent low expression levels (less than 10% of the main gene product). Different colors and length in the N or *C* terminus represent distinct sequences and number of AA compared to the main isoform (e.g., ERK1c). In order to make changes more visible, the scale is not always accurate. * insertion, ** β2 without insertion, γ2 with insertion, *** α1/α2 have different length *C* termini, # alternative exon 6 between the α and β isoforms that result in a change of 5–7 amino acids in this region. ## deletion. More information on the structure appears in Table 1.

Mitogen-activated protein kinases (MAPKs) are a group of signaling proteins that regulate almost all stimulated cellular processes, including proliferation, differentiation and stress response [1,2]. Dysregulation of these kinases is involved in many pathologies such as cancer, inflammation, developmental disorders and neurological diseases [3,4,5,6]. The signals to the MAPKs are transmitted via sequential phosphorylation and activation of protein kinases operating within a linear signaling cascade. Four such cascades have been identified thus far, and these are named according to the components of their MAPK tiers (Figure 1): (i) extracellular signal-regulated kinase 1/2 (ERK1/2, MAPK1/3 [7,8,9,10]); (ii) c-Jun-*N*-terminal kinase 1-3 ((JNK1-3, MAPK8/9/10 [11,12,13]); (iii) p38MAPKα–δ ((p38α–δ, MAPK14/11/12/13 [14,15,16]); and (iv) ERK5 (BMK1, MAPK7 [17,18]) cascades. While MAPK-like proteins such as ERK3/4, ERK7/8 exist as well, they are not considered as genuine MAPKs because of their distinct mode of regulation [19]. The MAPK cascades are all activated and transmit the signals of most extracellular stimuli to regulate the corresponding cellular processes. However, each cascade preferentially regulates some processes in which the other cascades play only minor functions. Thus, the ERK cascade preferentially regulates proliferation, differentiation and migration, p38 regulates mainly stress and immune responses, JNK preferentially regulates stress response and apoptosis, and ERK5 is mainly involved in stress response as well as proliferation. In pathologies, ERK is mainly involved in cancer, p38 in inflammation, and JNK in neurodegenerative diseases [3]. Although each of these seemingly linear cascades uses similar mechanisms to transmit their signals, their output is dramatically different in response to various stimuli. The initial specificity that determines cell fate is mediated by the MAPKK to MAPK tiers, but the cascades are tightly regulated by other means as well. Many specificity-dictating processes have been identified [20] and widely reviewed including: (i) intensity and duration of the signals [21]; (ii) scaffolding interactions that bring components to close proximity and determine localization [22]; (iii) crosstalk with other signaling pathways [23]; (iv) substrate-related regulation [24]; and (v) dynamic subcellular localization of the MAPKs [25], which includes stimulated translocation [26,27]. However, the existence of different components in each tier of the cascades that may extend signaling specificity and functionality [1] has not been properly examined. These components are products of distinct genes, and alternatively spliced isoforms of the same genes. The alternative splicing is a product of either intron retention or exon skipping during the splicing event of the initially transcribed full gene transcript [28,29]. This is a conserved process in higher organisms and plants [30]. Thus, the products that are formed differ from each other by having either additional or less amino acid stretches in certain regions of the protein, and therefore, may vary in activity, binding or localization. In this review, we focus on the role of distinct MAPKK and MAPK gene products at the protein level, including their alternative spliced forms, in extending signaling specificity and determining the proper cell fate in response to distinct stimulations.

## 2. Multiple Isoforms Extend Signaling Specificity of the MAPK Cascades

One important way to extend the functions of the MAPK signaling cascades is through the existence of various components with distinct functions or regulations within each tier of the cascades. This is mostly apparent in the MAP3K tier of the four cascades, in which 2–20 distinct protein kinases may transmit distinct signals in response to varying stimulations. Unlike the considerable number of components in the upper tiers, the number of components in the MAPKK and MAPK tiers is more limited, encoded by one to four genes that in some cases may have several protein products generated by alternative splicing ([31], Figure 1, Table 1). For example, in the case of the ERK1, p38α and ERK5, each gene has one main product and additional, lowly expressed, alternatively spliced isoforms. JNK1-3 are somewhat different, as each gene has several alternatively spliced protein products with substantial expression, which varies between cell lines. Overall, it is thought that the main MAPKKs and MAPKs components in each cascade, which are very similar to each other, funnel the signals towards their downstream targets without allowing much fluctuation. Nevertheless, in some cases, even minor sequence differences between these main components are sufficient to cause different activities (e.g., MEK1/2 that differ in their Pro-rich domain, as well as the N and *C* termini [32]). Moreover, their specificity and functionality are extended by the minor alternative spliced isoforms, and the expression levels of the main components.

The signaling outcome of the different components in each tier of each cascade may also be regulated by their cognate expression levels. This point became apparent in the ERK components in knockout mice [53,54]. Whereas knockout of ERK1 has very little effect on mice survival [55], the knockout of ERK2 is embryonic lethal due to placental defects and defective mesoderm differentiation [56,57]. It was later shown that this is due to expression levels in the early stages of development [58,59]. Similar expression-related changes were shown for other MAPKKs and MAPKs (e.g., MEK1/2, or p38α/β [53,54]), but not in the JNK cascade, in which the small differential effects are mainly due to distinct activities and regulation of the components [60,61]. Importantly, in the ERK and p38 cascades, it is clear that the KO mice lack not only the main isoforms, but also the minor alternatively spliced ones. This, as well as the low expression levels and lack of sequence conservation among species initially raised questions about the function of the minor forms [62]. However, the accumulating data described below suggest that these forms do appear in many cell types and tissues, and may participate in unique signaling processes that are not essential for embryonic development [33]. It is possible that in knockout cells, the activity of these minute isoforms is compensated for by other components. Thus, despite the assumed linearity of the MAPKK-MAPK tiers, under some conditions they disseminate the signal to extend the signaling repertoire. Therefore, here we provide a detailed review of the redundant and unique roles of the MAPKK and MAPK in JNK, p38 and ERK cascades. We do not, however, cover the ERK5 cascade due to its limited number of components [63,64,65].

## 3. Redundant and Unique Roles of MAPKKs and MAPKs of the JNK Cascade

The MAPKKs of the JNK cascade are MKK4 (that also phosphorylates p38s), and MKK7, which have two [66] and six [39] spliced isoforms, respectively. They have one (MKK4) or two (MKK7) main isoforms, while the other spliced isoforms are less abundant, and the evidence for their protein expression is still limited. The main isoforms are similar to each other in their sequence as well as in their mode of activation by MAP3Ks. However, despite their sequence similarity (~75%), the main isoforms of MKK4 and MKK7 have distinct features that result in different activities. In particular, while MKK7 is specific to JNKs, MKK4 can also phosphorylate p38 and induce its full activation [67]. Moreover, the rate of phosphorylation of the Tyr and Thr residues within the JNKs is significantly different. MKK4 favors phosphorylating the Tyr within the TPY domain, while MKK7 preferentially phosphorylates the Thr residue [68]. This suggests that the full activation of JNKs in vivo may sometimes require a combined phosphorylation by the two MKKs. Interestingly, the knockout of each of the two genes is embryonic lethal, supporting the suggestion that they have unique functions [69,70,71]. Aside from the main isoforms, alternatively spliced isoforms may provide additional regulation. Thus, MKK4δ [66] is substantially (although less than the main MKK4 isoform) expressed in most mammalian tissues and induces cell proliferation rather than preventing it as the main isoform. As mentioned above, MKK7 has six isoforms, composed of three spliced isoforms in the *N* terminus (α−γ) and two in the *C* terminus, named as 1 or 2 [39]. In different cells, there might be different spliced isoforms of MKK7 that serve as a main JNK activating kinase (>85% of the gene products), which is typically either MKK7γ1 [40,72] or MKK7β1 [42]). The expression of MKK7β2 and γ2 is usually somewhat lower, while MKK7α1/2 are lowly expressed. Besides their differences in expression, MKK7 isoforms also differ in their activity (MKK7α1/2 are less active), and in their ability to bind scaffold proteins (e.g., Filamin [73] or JIP1 [74]). Finally, during T cell activation, MKK7 splicing is mediated by CELF2, which favors the MKK7γ1 isoform. This process is positively regulated by JNK, and thus consists a positive feedback loop upon stimulation [75].

The MAPK components in the JNK cascades, JNK1 and JNK2, are each transcribed into four confirmed transcripts, and JNK3 has three highly similar alternatively spliced isoforms [49,50]. The alternatively spliced isoforms of JNK1 and JNK2 result from either the inclusion of alternative sequences in intron 6 (either α or β), or different exons in the *C*-termini known as 1 or 2 [49,76]. The alternatively spliced isoforms of JNK3 have either elongated *N* or *C* termini ((JNK3α1(L), JNK3α2(L) or (JNK3α2(S)), which give rise to different size proteins. Other alternative spliced isoforms of these genes are transcribed into RNA but have not been found in the protein level. Interestingly, only double-knockout mice of JNK1 and JNK2 are embryonic lethal due to severe brain malformation, while the knockout of each one alone, or together with JNK3, results in viable animals with limited metabolic, immunological, and other pathologies [77,78]. Moreover, each one of the JNK proteins has similar catalytic activity towards most substrates, such as Ser34 of p53 [79], indicating that JNK1, JNK2, and to some extent JNK3 are mostly functionally redundant. However, the relative protein expression of JNK1 and JNK2 isoforms varies between tissues and cells, though all are ubiquitously expressed, while JNK3 is expressed almost exclusively in the brain and testis. The variable expression contributes to the limited differences in the single knockout animals, which results mostly from distinct signaling specificity exerted by tissue-specific substrates and interactions [50,80,81]. Among other outcomes, JNK1 knockout mice demonstrate a dysregulated neuronal differentiation during development [82], while JNK3 knockout effects on the developing brain are more limited, showing mainly hippocampal neurogenesis malformations [83,84]. JNK2 does not seem to participate in these processes, but plays a major role in carcinogenesis [85]. In addition, there are some cases in which the actions of JNK1 and JNK2 are cooperative or even synergistic, such as the development of skin keratinocytes [86] or UV- and arsenite-induced apoptosis [87]. As for the alternatively spliced forms of JNK2, it was shown that JNK2α3 has higher activity towards the transcription factor AP-1 than JNK2β3 [88]. Another study revealed that the short JNK proteins are less stable than the long isoforms due to the interaction of the latter with the scaffold protein JIP1 [89]. This stability modulation represents a new mechanism to regulate the JNK pathway. Thus, it is clear that the different JNK components can provide extended specificity to the cascade in response to various stimulations.

## 4. Redundant and Unique Roles of MAPKKs and MAPKs of the p38 Cascade

The MAPKKs of the p38 cascade are MKK3, MKK6, and occasionally also MKK4. Each of them has two alternatively spliced isoforms that are substantially expressed, but the expression level of the main isoforms (MKK3/MKK6), although varied between cells, are always higher than the expression of the other (MKK3b/6b) [35,37]. The human MKK3 and MKK6 are similar in their primary sequences (~ 80%), and in their ability to phosphorylate the TGY motifs of a given p38 protein [37]. However, each is activated by distinct stimuli [37], and preferentially (but not exclusively) phosphorylate distinct p38 isoforms [90,91,92]. In addition, it was shown that the balance between MKK3 and MKK6 mediates p38-associated resistance to cisplatin [93]. During development, MKK3/6 are mostly functionally redundant, as knockout of each of them individually does not affect viability, while simultaneous knockout of both is embryonic lethal due to problems in the hematopoietic system [94]. Interestingly, MKK3 and MKK6 have process, or cell type-specific, functions. For example in the differentiation of T cells, the individual knockouts yielded aberrant T cell maturation and function [95], but those differences are not sufficient to induce embryonic lethality. No particular differences in activity have been demonstrated for the distinct alternatively spliced isoforms, and although the main isoforms are the most active in most cases, MKK3b and 6b may also serve as the main p38α−δ activators under some conditions [38,96].

Four p38 MAPKs genes (α−δ) have been identified in humans, p38α has ~80% identity to p38β (also known as p38β2), while p38γ has ~80% identity to p38δ, but the p38α/β pair has just 60% identity to the p38γ/δ pair [14,97]. The only spliced variants reported are of p38α (Exip and Mxi2), which are both lowly expressed. Interestingly, all of the main gene-products are able to phosphorylate similar substrates and share similar regulation. However, in some cases they display distinct properties and differ in their substrate specificity. For example, it was shown that p38α/β phosphorylate MK2 and MK3 better than p38γ/δ [98], while the latter phosphorylate the tau protein better [99]. These changes are probably due to differences in their substrate binding. While p38α is ubiquitously expressed, the expression of the other three isoforms varies or is even restricted to specific tissues. Knockout of p38α is embryonic lethal due to placental defects [100], while p38β−δ knockout mice are viable and fertile [101,102]. The different effects may be mediated by distinct expression of the isoforms or by a difference in substrate repertoire. The specificity of the p38 is also extended by the alternatively spliced proteins of p38α. The first confirmed alternatively spliced form of p38α was named Exip (exon skip), whose *C* terminus is modified, and it is considerably less abundant than the main p38α isoform [44,45]. Exip is not phosphorylated on its activatory TGY motif due to lack of MAPKKs binding sites, but it can induce earlier apoptotic onset upon stress or by binding to a Toll receptor. The second lowly expressed spliced variant is Mxi2 (Max interactor), which affects the c-Myc transcriptional activity [46]. It has a unique 17 amino acids in subdomain XI, and lacks the whole *C*-terminus [47]. Mxi2 is usually poorly expressed, but it is found in relatively high amounts in mouse kidney where its expression level is reduced by ischemia [103]. It was also shown that Mxi2 interacts with ERK1/2, regulating their non-stimulated nuclear translocation and sustaining their nuclear phosphorylation [48,104,105]. Interestingly, it was recently shown that Mxi2 is highly expressed in prostate cancer, where it increases the aggressiveness of the disease, acting mainly by interacting with the Argonaut2/mir1285 complex that further regulate p53 activity [106]. Moreover, osmotic stress induces p38-dependent alterative splicing by inducing p38 activation and phosphorylation of the spliceosome component SKIIP [107]. Phosphorylated SKIIP phosphorylation then modulates the alternative splicing of GADD45α, which is an upstream activator of the p38 pathway. These findings suggest that the phosphorylation of SKIIP by p38 forms a feedback loop that regulates various alternative splicing events.

## 5. The MEK1b-ERK1c Axis

The main proteins translated by the ERK1 and ERK2 genes are the 44 and 42 kDa isoforms, respectively. These isoforms usually share similar characteristics, although some differences between them do exist under restricted conditions or in specific cell types [108,109,110,111,112,113,114,115,116,117]. Aside from these main isoforms, initial studies reported the existence of additional, slightly different transcripts called ERK1psi [118] and ERK2a [119]; however, these transcripts exist only on the RNA level and are not translated into a stable protein. On the other hand, the use of pan-ERK antibodies revealed an additional band of 46 kDa in rat-derived cells. This band was not initially cloned and was tentatively labeled ERK4 [118]. Later on, the band was also found in mice, but not in humans, and was further studied by our group. This latter study was based on in-gel kinase assay of rat cells that aimed to identify elevated kinase activity in transformed cells. One phosphorylated band at 46 kDa stood out as the most notable kinase activity, and was even increased after Ras transformation. This band was purified and cloned, giving rise to an alternatively spliced isoform of ERK1 with a 26 amino acid insertion (intron 7) inside the common docking motif (CD) of ERK1 of rat and mouse. The expression of this isoform was much lower than that of ERK1 and ERK2, consisting of 1–10% of the total rodent ERK proteins. Further characterization confirmed the similarity of this protein to the band originally identified as ERK4, and therefore it was renamed ERK1b. Under many circumstances, ERK1b behaves similarly to ERK1/2, but in Ras-transformed Rat1 cells, the expression level of ERK1b is elevated, and it is phosphorylated in a different kinetic than the main isoforms. Additionally, ERK1b was shown to be the major isoform that transmits extracellular signals in transformed cells. The reason for the distinct regulation is the alteration in the CD domain, which results in distinct binding to scaffolds, substrates, activators and phosphatases.

The lack of 46 kDa band in other organisms prompted a study that aimed to identify whether any alternative spliced isoform is generated in humans. Using PCR, Aebersold et al. demonstrated that inclusion of ERK1′s intron 7 does occur in human as well, although the amount of expression may vary between cell types [43]. However, since the intron contains an in-frame stop codon in humans, the translation of this isoform resulted in a 42 kDa protein. This splicing event alters the C-terminal, containing 18 unique AA sequence in the very *C* terminus of the protein that is different than the CD domain of ERK1. Since the primate’s alternatively spliced isoform was different from the rodent ERK1b, it was named ERK1c. This protein migrates to the same place as ERK2 on SDS-PAGE, and therefore cannot be detected by pan-ERK antibodies. However, the expression of this protein was confirmed using specific antibody to the unique ERK1c sequence, which detected low expression (~10 of ERK1) in various human cells [43]. Unlike the diffused subcellular localization of ERK1/2, ERK1c was found localized all over the cytoplasm in some cells, while in G2/M cells, it was localized primarily in the Golgi. In depth study of this phenotype revealed that ERK1c regulates Golgi fragmentation, which was specifically prevented by expression of dominant-negative ERK1c. Moreover, the expression and activity of ERK1c is increased upon mitosis followed by its accumulation in the Golgi where it regulates mitotic Golgi fragmentation [120]. Indeed, small interfering RNA specific to ERK1c (with no effect on ERK1 or any other protein tested) significantly attenuated this process, whereas ERK1c overexpression facilitated it. These effects were also reflected in mitotic progression, indicating that ERK1c is involved in cell cycle regulation via modulation of Golgi fragmentation. The main isoforms of ERK1 and ERK2 are active in mitosis as well, but do not significantly replace ERK1c.

**Figure 2:**
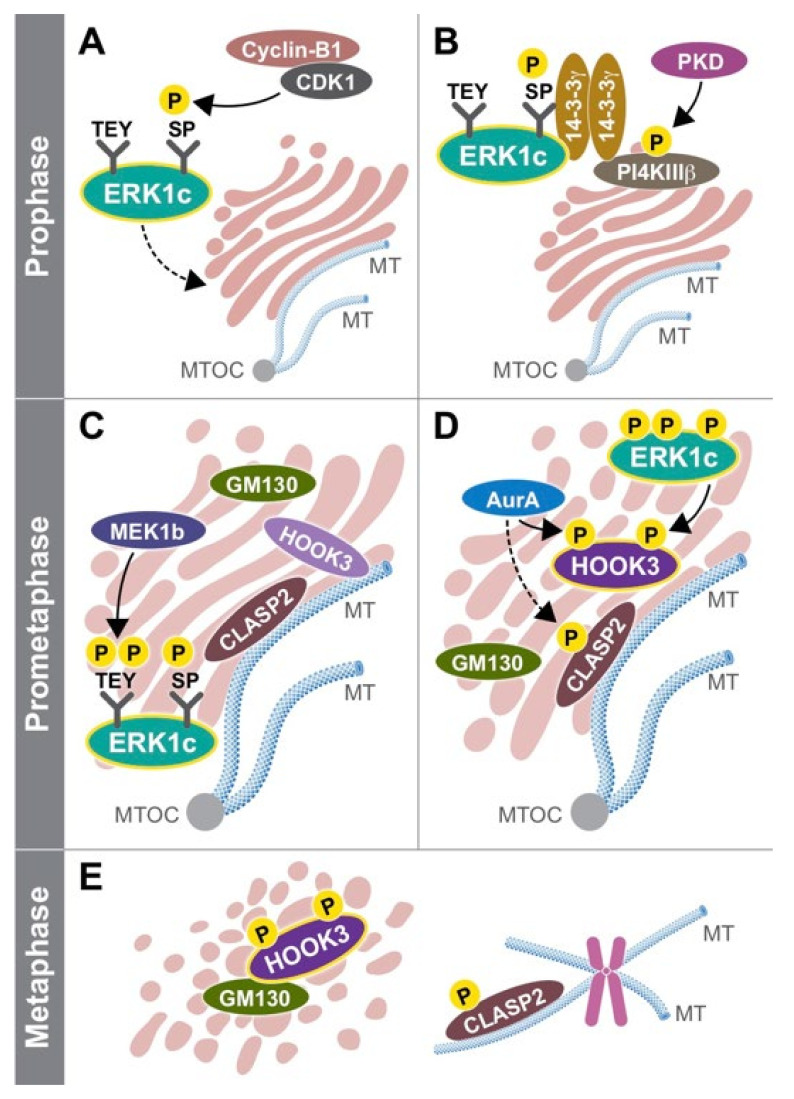
Schematic presentation of ERK1c function in the Golgi. An illustration demonstrating the unique role of ERK1c isoform in the Golgi fragmentation process at different stages of the cell cycle. **Prophase**: (**A**) CDK1 becomes active, and phosphorylates ERK1c on Ser343 in its unique *C*-terminus. (**B**) Phosphorylated ERK1c interacts with a shuttling complex of PI4KIIIβ and 14-3-3γ, which mediates its Golgi translocation. **Prometaphase:** (**C**) In the Golgi, ERK1c is phosphorylated by MEK1b, becomes fully active and induces mitotic Golgi fragmentation. The Golgi is organized in stacks, while GM130 is found in between these stacks. The microtubules (MTs) are polymerized from the microtubules originating center (MTOC) and stabilize the Golgi structure. Both HOOK3 and CLASP2 interact together with the MTs and Golgi. (**D**) Once entering mitosis, HOOK3 is phosphorylated by both ERK1c and AurA, while CLASP2 might be phosphorylated by AurA. At this time the Golgi stacks starts to break into ribbons. **Metaphase**: (**E**) The phosphorylation of HOOK3 and CLASP2 allow the complete fragmentation of the Golgi into a haze. At this time, phosphorylated CLASP2 maintains its interaction with the MTs that originate from the centromeres, while phosphorylated HOOK3 interacts with GM130.

One question that was unsolved at this stage was the way in which ERK1c is activated. This question was raised due to the replacement of the residues just C-terminal to the CD that suggest a reduced interaction with the upstream activators MEK1 and MEK2. Using the three MEK constructs in a different study, Shaul et al. found that ERK1c is preferentially activated by MEK1b and not by MEK1 or MEK2 [33]. Interestingly, MEK1b was initially considered an inactive isoform, because it was unable to phosphorylate the main isoform of ERK1 and ERK2 [121]. Apparently, these results indicate that this alternatively spliced isoform has a unique specificity towards ERK1c. Additionally, Shaul et al. showed that MEK1b phosphorylation and activity are preferentially stimulated by mitotic inducers (e.g., nocodazole) rather than growth factors or stress mediators, to induce its specific activity toward ERK1c in mitosis (Figure 2). MEK1/2, on the other hand, preferentially target ERK1/2 and not ERK1c in response to mitogens, without affecting the Golgi [122]. Importantly, similarly to ERK1c, MEK1b expression and activity are elevated during mitosis, though this isoform is localized primarily in the Golgi throughout the cell cycle. These results indicate that the ERK cascade can be divided into two routes: the classic MEK1/2–ERK1/2 route that mediates most extracellular signals, and the splice-variants MEK1b–ERK1c route that specifically regulates mitotic Golgi fragmentation, and thus extends the specificity of the ERK cascade.

Since ERK1c is localized in the cytoplasm of cycling cells, Wortzel et al. studied the molecular machinery that regulates its translocation to the Golgi during mitosis. It was found that this translocation is mediated at the prophase and prometaphase, and requires CDK1-induced phosphorylation of Ser343 of ERK1c [123,124]. This phosphorylation then facilitates binding of phosphorylated ERK1c to a protein complex of PI4KIIIβ and 14-3-3γ. Interestingly, the stability of this complex is regulated by protein kinase D (PKD)-mediated phosphorylation of PI4KIIIβ. Thus, ERK1c joins a small group of other Golgi shuttling proteins at G2 to M phase transition that integrate Golgi-regulating processes such as fragmentation into one coherent pathway. To better understand the function of ERK1c, Wortzel et al. recently studied the ERK1c-specific substrates that regulate Golgi fragmentation [125]. By screening several putative phosphorylated Golgi proteins, HOOK3 was identified as a mediator of ERK1c-induced Golgi fragmentation. ERK1c phosphorylates HOOK3 on Ser238, which is a prerequisite for an additional phosphorylation on Ser707 by AuroraA kinase. In cycling cells, HOOK3 interacts with microtubules (MTs) and links them to the Golgi to induce the stabilization of the organelle. During mitosis, the double phosphorylation of HOOK3 by ERK1c and AuroraA allows the detachment of HOOK3 from the MTs, and its interaction with the Golgi architectural protein GM130. This switch in HOOK3′s interactions reduces the Golgi stability, which permits Golgi fragmentation. Thus, ERK1c supports the Golgi fragmentation process by shuttling into the Golgi towards mitosis, where it is activated by the resident MEK1b. The active ERK1c then phosphorylates several substrates including HOOK3 to set the stage for the fragmentation.

## 6. Conclusions

The MAPK cascades are central pathways that disseminate the signals of essentially all extracellular stimuli. Although all these cascades use a similar mechanism to transmit the signals, each cascade leads to a variety of distinct downstream activities and cellular fate. These differences raise the question of signaling specificity, and this review demonstrates that despite the similarity of the components of each tier in each cascade, they have substantial variability that extends the signaling repertoire, and allows each stimulation to secure the proper cell fate.

Many studies have suggested that the components in each tier of each MAPK cascade are very similar to each other and may have redundant effects. The evidence presented in this review clearly shows that despite this similarity, the different components present distinct expression and activities that make the cascades less linear than expected. An important example shown here is the MEK1b–ERK1c axis, which presents a separate signaling route with distinct activity compared to the main ERK isoforms. More studies on the activities of the distinct isoforms of each MAPK cascade component, including the alternatively spliced isoforms are required to expand our understanding of the signaling specificity of the MAPK cascades. As for the MEK1b–ERK1c axis, it seems to specifically regulate cell cycle progression, and therefore, may be used as a clinical target for cancer and other diseases. Similarly, the involvement of Mxi2 in regulating prostate cancer should make it a specific target for this disease. Therefore, these two isoforms should be investigated in detail, mainly by isolating additional specific substrates of ERK1c or Mxi2 to determine their mechanism of action in health and disease. Thus, alternatively spliced isoforms extend signaling specificity, and are essential for decisions regarding the proper cell fate. Further studies are required to understand their exact roles and how they can be utilized as clinical targets.

## Figures and Tables

**Table 1:** Characteristics of the alternative spliced isoforms of the components of the MAPKKs and MAPKs of all four MAPK cascades. The alternatively spliced isoforms presented are only from proteins whose expression has been confirmed, although more lowly expressed forms whose transcripts have been identified may exist. The structural and functional properties of the lowly expressed alternatively spliced isoforms of the ERK1/2, p38, and ERK5 cascades are presented as compared to the main isoforms. These main isoforms are not presented here as their properties are very well covered in many other reviews. As for the components of the MAPKK tier of the JNK and p38 cascade, the alternatively spliced isoforms are presented as compared to the main isoforms MKK3, MKK6, MKK4 and MKK7γ1 (not presented), whose expression is usually higher than the others, although the latter might be substantially expressed in some cells. Due to similar expression level of several JNKs, there is no “main” isoform and the comparison of the spliced isoforms is to the other components in the tier.

	Alternative Spliced Forms	Product of:	Sequence Changes	MW	Functional Changes	Ref.
**MAPKKs**	MEK1b	MAP2K1 (MEK1)	Deletion of 26 AA in subdomain 5 of MEK1.	43.5 kDa	Reduced activity, change in substrate specificity.	[33,34]
	MKK3b	MAP2K3 (MKK3)	Additional 29 amino acids N-terminal to MKK3.	40 kDa	Slightly Elevates substrates’ phosphorylation.	[35,36]
	MKK6b	MAP2K6 (MKK6)	Additional 56 amino acids N terminal to MKK6.	38.5 kDa	Affect substrate specificity.	[37,38]
	MKK7α1	MAP2K7 (MKK7)	Deletion of the N terminal 89 AA of MKK7γ1.	40 kDa	Reduced activity due to lack of JNK binding domain.	[39]
	MKK7α2	MAP2K7 (MKK7)	Deletion of the N terminal 89 AA, + additional 33AA in the *C* terminus, compared to MKK7γ1.	43.5 kDa	Reduced activity due to lack of JNK binding	[39]
	MKK7β1	MAP2K7 (MKK7)	Deletion of 16 amino within the *N* terminus as compared to MKK7γ1.	50 kDa	Distinct subcelluar localization, and lower binding to JNK due to lack of D domain. Distinct effect in cancers compared to MKK7γ1	[39,40,41,42]
	MKK7β2	MAP2K7 (MKK7)	Deletion of 16 amino within the *N* terminus + addition of 33 AA to the *C* terminus compared to MKK7γ1.	53.5 kDa	Distinct subcelluar localization, a lower binding to JNK and distinct effect in cancers compared to MKK7γ1	[39,40,41,42]
	MKK7γ2	MAP2K7 (MKK7)	Addition of 33 AA to the *C* terminus, as compared to MKK7γ1.	53.5 kDa	No known differences	[39]
**MAPK**	ERK1c	MAPK1 (ERK1)	Change of the C terminal 40 AA with 18 other AA compared to ERK1.	41.5 kDa	Reduced activity and change of substrate specificity as compared with ERK1.	[33,43]
	Exip	MAPK14 (p38α)	Change of the C terminal 106 AA with 53 other AA compared to p38α.	35.5 kDa	Reduced activity. Change in subcellular localization and protein interaction, as compared to p38α	[44,45]
	Mxi2	MAPK14 (p38α)	Change of the C terminal 81 AA with 17 other AA compared to p38α.	34 kDa	Reduced activity, change in substrate specificity, distinct protein interaction, and distinct regulation as compared to p38α.	[46,47,48]
	JNK1α1	MAPK8 (JNK1)	Alternative exon 6a, Short *C* terminus	46 kDa	Alternative substrate binding compared to β1 and β2.	[49,50]
	JNK1α2	MAPK8 (JNK1)	Alternative exon 6a, long *N* terminus (43 AA)	54 kDa	Alternative substrate binding compared to β1 and β2.	[49,50]
	JNK1β1	MAPK8 (JNK1)	Alternative exon 6b, short *C* terminus	46 kDa	Changes in expression level from α1 and α2.	[49,50]
	JNK1β2	MAPK8 (JNK1)	Alternative exon 6b, long *N* terminus (43 AA)	54 kDa	Changes in expression level from α1 and α2.	[49,50]
	JNK2α1	MAPK9 (JNK2)	Alternative exon 6b, short *C* terminus	46 kDa	Alternative substrate binding compared to β1 and β2.	[49,50]
	JNK2α2	MAPK9 (JNK2)	Alternative exon 6b, long *C* terminus (42 AA)	54 kDa	Alternative substrate binding compared to β1 and β2.	[49,50]
	JNK2β1	MAPK9 (JNK2)	Alternative exon 6a, short *C* terminus	46 kDa	Alternative substrate binding compared to α1 and α2.	[49,50]
	JNK2β2	MAPK9 (JNK2)	Alternative exon 6a, long *C* terminus (42 AA	54 kDa	Alternative substrate binding compared to α1 and α2.	[49,50]
	JNK3α1(L)	MAPK10 (JNK3)	Long *N* terminus (38 AA) short *C* terminus.	50.5 kDa	Probable changes in protein interaction.	[49,50,51]
	JNK3α2(L)	MAPK10 (JNK3)	Long *N* terminus (38 AA) Long *C* terminus (42 AA).	58.5 kDa	Probable changes in protein interaction.	[49,50,51]
	JNK3α2(S)	MAPK10 (JNK3)	Short *N* and long *C* termini (42 AA).	54 kDa	Probable changes in protein interaction.	[49,50,51]
	ERK5-T	MAPK7 (ERK5)	Change of the *C* terminal 324 AA with 41 other AA.	61 kDa	Distinct subcellular localization and protein binding compared to ERK5.	[52]

## Data Availability

All data appear in the review.
